# The genomes of two parasitic wasps that parasitize the diamondback moth

**DOI:** 10.1186/s12864-019-6266-0

**Published:** 2019-11-21

**Authors:** Min Shi, Zhizhi Wang, Xiqian Ye, Hongqing Xie, Fei Li, Xiaoxiao Hu, Zehua Wang, Chuanlin Yin, Yuenan Zhou, Qijuan Gu, Jiani Zou, Leqing Zhan, Yuan Yao, Jian Yang, Shujun Wei, Rongmin Hu, Dianhao Guo, Jiangyan Zhu, Yanping Wang, Jianhua Huang, Francesco Pennacchio, Michael R. Strand, Xuexin Chen

**Affiliations:** 1 Institute of Insect Sciences, College of Agriculture and Biotechnology, Zhejiang University, Hangzhou, 310058 China; 2 Ministry of Agriculture Key Lab of Molecular Biology of Crop Pathogens and Insect Pests, Zhejiang University, Hangzhou, 310058 China; 3 State Key Lab of Rice Biology, Zhejiang University, Hangzhou, 310058 China; 4 BGI-Tech, BGI-Shenzhen, Shenzhen, 518083 China; 5 Xingzhi College, Zhejiang Normal University, Jinhua, 321000 Zhejiang China; 6 School of Physical Science and Technology, Shanghai Tech University, Shanghai, 201210 China; 7 Institute of Plant and Environmental Protection, Beijing Academy of Agriculture and Forestry Sciences, Beijing, 100097 China; 8 Dipartimento di Agraria, Laboratorio di Entomologia “E. Tremblay”, Università di Napoli “Federico II”, Via Università 100, 80055 Portici, NA Italy; 9 Department of Entomology, University of Georgia, Athens, GA 30602 USA

**Keywords:** *Cotesia vestalis*, *Diadromus collaris*, Parasitic wasps, Genome, Transcriptome

## Abstract

**Background:**

Parasitic insects are well-known biological control agents for arthropod pests worldwide. They are capable of regulating their host’s physiology, development and behaviour. However, many of the molecular mechanisms involved in host-parasitoid interaction remain unknown.

**Results:**

We sequenced the genomes of two parasitic wasps (*Cotesia vestalis*, and *Diadromus collaris*) that parasitize the diamondback moth *Plutella xylostella* using Illumina and Pacbio sequencing platforms. Genome assembly using SOAPdenovo produced a 178 Mb draft genome for *C. vestalis* and a 399 Mb draft genome for *D. collaris*. A total set that contained 11,278 and 15,328 protein-coding genes for *C. vestalis* and *D. collaris*, respectively, were predicted using evidence (homology-based and transcriptome-based) and de novo prediction methodology. Phylogenetic analysis showed that the braconid *C. vestalis* and the ichneumonid *D. collaris* diverged approximately 124 million years ago. These two wasps exhibit gene gains and losses that in some cases reflect their shared life history as parasitic wasps and in other cases are unique to particular species. Gene families with functions in development, nutrient acquisition from hosts, and metabolism have expanded in each wasp species, while genes required for biosynthesis of some amino acids and steroids have been lost, since these nutrients can be directly obtained from the host. Both wasp species encode a relative higher number of neprilysins (NEPs) thus far reported in arthropod genomes while several genes encoding immune-related proteins and detoxification enzymes were lost in both wasp genomes.

**Conclusions:**

We present the annotated genome sequence of two parasitic wasps *C. vestalis* and *D. collaris*, which parasitize a common host, the diamondback moth, *P. xylostella*. These data will provide a fundamental source for studying the mechanism of host control and will be used in parasitoid comparative genomics to study the origin and diversification of the parasitic lifestyle.

## Background

Parasitic insects, particularly the parasitic wasps, are a large group of animals [1–4]. As adults, most species feed on nectar, while larvae feed as parasites on other arthropods. Adult females of parasitic wasps usually lay their eggs on or inside the body of a host, which usually dies when offspring complete their development [2, 3]. Parasitic wasps are major natural enemies of a vast number of arthropod species in many orders [4]. Many species are also widely used as biological control agents of pests in agricultural and forest ecosystems [5, 6]. Most wasps have narrow host ranges, successfully develop in only one or a few species, and also parasitize only one life stage of their host (egg, larva, pupa, or adult) while many wasps share a common host species. Parasitic wasps that lay their eggs on hosts usually produce progeny that feed as ectoparasites, while species that lay their eggs in hosts produce progeny that feed as endoparasites [7]. Parasitic wasps are also either solitary, producing a single offspring per host, or gregarious and produce multiple offspring per host [7]. Parasitic wasps usually produce a number of virulence factors following oviposition that benefit offspring by altering the growth, development and immune defenses of hosts. The sources of these virulence factors include venom [8], symbiotic polydnaviruses (PDVs) [9–12], and teratocytes [13].

**Fig. 1:**
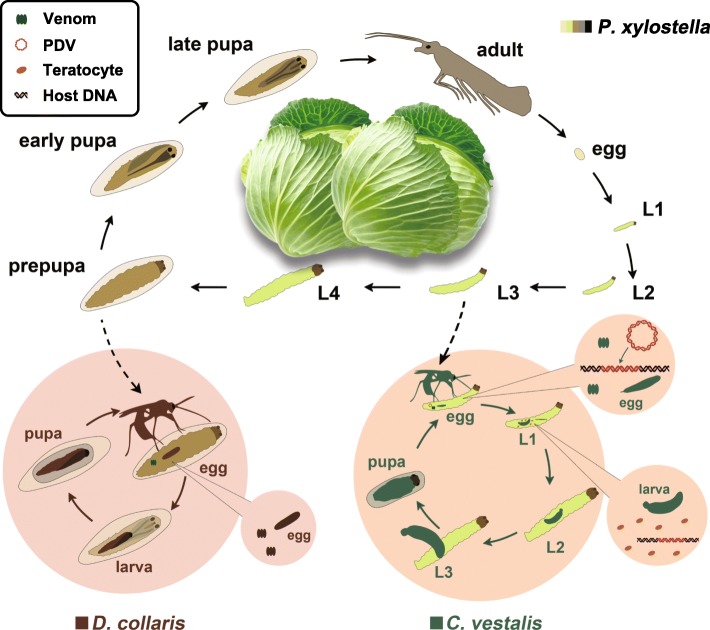
The life history of *C. vestalis* and *D. collaris*. *C. vestalis* preferentially parasitizes second and third instar *P. xylostella* larvae (L2 and L3); and *D. collaris* parasitizes pupal stage hosts

The genomes of more than 15 parasitic wasp species that parasitize different hosts have been sequenced (www.ncbi.nlm.nih.gov). These include *Nasonia vitripennis* (Hymenoptera: Pteromalidae), which is an ectoparasitoid that parasitizes the pupal stage of selected Diptera [14], *Microplitis demolitor* (Hymenoptera: Braconidae), which is an endoparasitoid that parasitizes the larval stage of selected species of Lepidoptera [15], and *Fopius arisanus,* which is an endoparasitoid that parasitizes larval stage Diptera in the family Tephritidae [16]. Collectively, these data provide several insights into parasitoid wasp biology. In contrast, no studies have examined the genomes of different species that parasitize the same host. Here, we sequenced two endoparasitoids in the superfamily Ichneumonoidea that parasitize the diamondback moth, *Plutella xylostella* L. (Lepidoptera: Yponomeutidae), which is a major worldwide pest of cruciferous crops (Fig. 1) [17, 18]. *Cotesia vestalis* (Haliday) is a solitary, larval endoparasitoid in the family Braconidae (Braconidae: Microgastrinae) that produces venom, a PDV named *C. vestalis* bracovirus (CvBV) and teratocytes. Larvae of *P. xylostella* parasitized by *C. vestalis* exhibit greatly reduced weight gain, delayed larval development and disabled cellular and humoral immune defences [19–21]. *Diadromus collaris* (Gravenhorst), is in the family Ichneumonidae (Ichneumonidae: Ichneumoninae) and is a solitary pupal endoparasitoid. *D. collaris* produces only venom. *P. xylostella* pupae parasitized by *D. collaris* fail to develop into adults and exhibit suppressed humoral and cellular immune defences [21].

In this study, we present the annotated genome sequence of two parasitic wasps *C. vestalis* and *D. collaris*, which parasitize a common host, the diamondback moth, *P. xylostella*. The gathered genomic data and transcriptome datasets collected from varying developmental stage and tissues will significantly expand our comprehension of the evolutionary history of parasitic wasps and their interactions with the common host, *P. xyostella*.

## Results

### Genome assembly and gene information

The whole genome sequencing was performed by combining Illumina Solexa sequencing based on HiSeq 2000 platform (Illumina, San Diego, CA, USA) and the Long-Read Single Molecule Real-Time (SMRT) sequencing based on PacBio Sequel platform (Pacific Biosciences, Menlo Park, CA, USA) in consideration of cost and the low heterozygosity of wasp genome [22, 23]. In total, we obtained 36.10 Gb of raw data (32.05 Gb from Illumina platform and 4.05 Gb from PacBio platform) for *C. vestalis*, and 65.91 Gb of raw data (61.05 Gb from Illumina platform and 4.86 Gb from PacBio platform) for *D. collaris* (Additional file 1: Table S1). After filtering steps, 25.55 Gb (127.78×) from *C. vestalis* and 49.19 Gb (120.86×) from *D. collaris* were assembled using SOAPdenovo V2.04 [24] (Additional file 1: Table S2). These data were further assembled into a 178 Mb draft genome for *C. vestalis* and a 399 Mb draft genome for *D. collaris*, which were consistent with genome size estimates generated by k-mer analysis (Table 1; Additional file 1: Figure S1). Genome assemblies for *C. vestalis* and *D. collaris* yielded scaffold N50 s that were 2.60 Mb and 1.03 Mb, respectively (Additional file 1: Table S3). We then checked the distribution of sequencing depth against GC content to infer the abundance of potential contamination of bacteria. As for GC content, compared with *C. vestalis* (29.96%), *D. collaris* has a higher GC content, around 37% (Table 1, Additional file 1: Figure S2). The bacterial contaminant reads in genome data of *C. vestalis* (Additional file 1: Figure S2) were filtered out after the assembling procedure. All transcripts were mapped to genome assemblies by BLAT with default parameters, resulting 91.7% transcripts of *C. vestalis* and 98.1% of *D. collaris* were found in the assembled genome, respectively (Additional file 1: Table S4). The quality of the assembly was further checked by Benchmarking Universal Single-Copy Orthologs BUSCO v3.0.2 [25] with insectdbV9 as referenced dataset. The recovered genes are classified as ‘complete’ when their lengths are within two standard deviations of the BUSCO group mean length. BUSCO analysis indicated the complete recovered genes for each species was greater than 96.7% (Table 1). These metrics strongly supported the overall quality of genome assemblies.

**Table 1:** Assembled Genomes and Gene Sets for *C. vestalis* and *D. collaris*

	*C. vestalis*	*D. collaris*
Contig N50 (bp)	51,333	25,941
Scaffold N50 (Kb)	2609.601	1030.36
Quality control (covered by assembly)		
Genome size (Mb)	178.55	399.17
Number of scaffolds	1437	2731
BUSCO (*n* = 1658) (%)	C^a^: 96.7%, F: 2.4%	C: 99.2%, F: 0.3%
Genomic features		
Repeat (%)	24	37
G + C (%)	29.96	37.37
Gene annotation		
Number of genes	11,278	15,328

^a^C: complete BUSCOs; F: fragmented BUSCOs

**Fig. 2:**
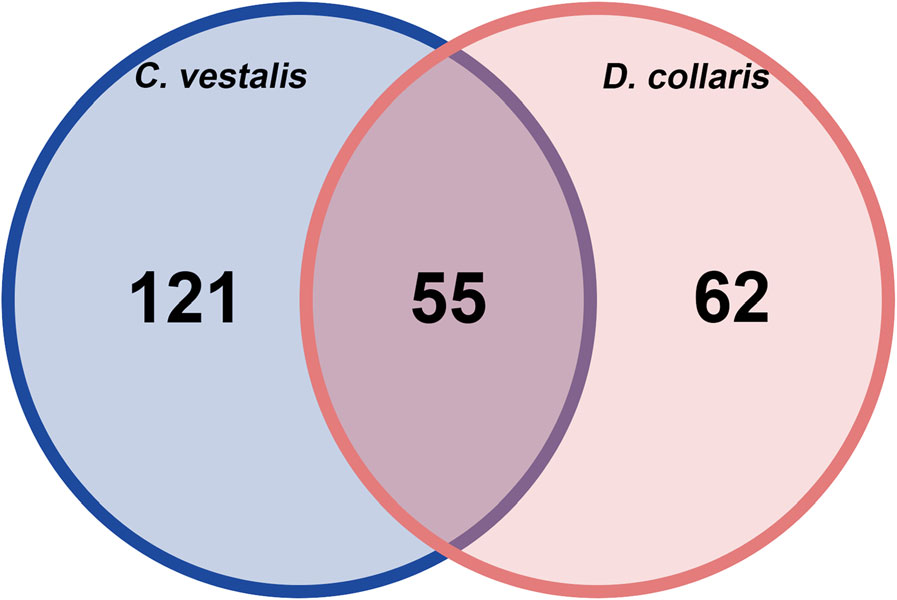
Venn diagram of the distribution of unique and shared miRNAs across *C. vestalis* and *D. collaris*. A final set of 176 miRNAs in *C. vestalis* and 117 miRNAs in *D. collaris*. Strikingly, 55 miRNAs were conserved in these two wasp genomes

A total of 11,278 protein-coding genes for *C. vestalis* and 15,328 for *D. collaris* were identified by de novo and evidence-based (homology-based and transcriptome-based) prediction methods (Table 1, Additional file 1: Table S5 and S6). About 85% of the inferred proteins for *C. vestalis* and 76.31% for *D. collaris* were annotated using the databases of KEGG, GO, TrEMBL, SWISS-PROT and InterPro (Additional file 1: Table S7). Gene numbers were higher than for *Apis mellifera* (10,660), but lower than for *N. vitripennis* (17,084). As estimated by homology-based and de novo prediction methods, repetitive DNA accounted in *D. collaris* genome assembly (37%) was higher than that in *C. vestalis* (24%) (Additional file 1: Table S8), indicating the partial reason for the larger genome size of *D. collaris.* The total size of transposable elements (TEs) approached 31.1 Mb (17.4% of genome) for *C. vestalis* and 119.5 Mb (29.93% of genome) for *D. collaris* (Additional file 1: Table S8). TE diversity in *D. collaris* is 17% higher than that in *N. vitripennis* (66 Mb, 22% of genome) and is 10-fold higher than that in *A. mellifera* (6.2 Mb, 2.8% of genome). We sequenced small RNA of these two wasps by constructing small RNA libraries and used prediction software to identify a final set of 176 miRNAs in *C. vestalis* and 117 miRNAs in *D. collaris*. Both numbers are relatively higher than those in *N. vitripennis* (98 miRNAs) and *A. mellifera* (94 miRNAs), but much lower than those in *D. melanogaster* (165 miRNAs) (Fig. 2 and Additional file 2: Table S9). Beside 55 known miRNAs that conserved across these two wasp genomes, we also identified total 47 novel, previously uncharacterized miRNA, 14 of them were specific to *C. vestalis* and the rest were specific to *D. collaris*. The small number of conserved miRNAs and relatively large number of novel miRNAs was somewhat surprising in light of each species developing in the same host and living in the same habitats where *P. xylostella* occurs.

### Genome phylogeny and comparisons

**Fig. 3:**
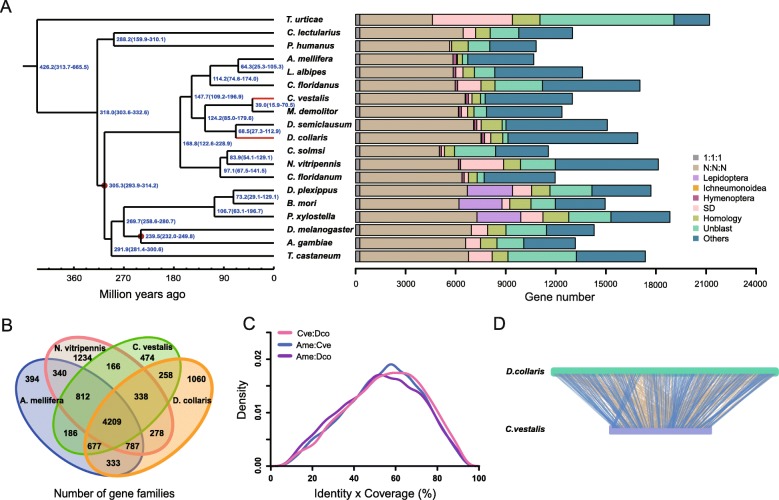
Phylogenetic tree and orthologue assignments of 19 arthropod genomes. **a** The phylogenetic tree was constructed from 262 single-copy genes using maximum likelihood methods. Red points on the internal nodes indicate fossil calibration times in the analysis. Blue numbers indicate estimated divergence times (Mya, million years ago). The red branches identify the two wasps sequenced in this study. The different types of orthologous relationships are shown. “1:1:1” = universal single-copy genes, although the absence in a single genome is tolerated; “N:N:N” = orthologues in all genomes, although the absence in less than 2 genomes is tolerated. “Ichneumonoidea” = ichneumonoid-specific genes, although the absence in a single genome is tolerated; “Lepidoptera” = lepidopteran-specific genes, although the absence in a single genome is tolerated; “SD” = species-specific duplicated genes; “Homology” = genes with an e-value less than 1e-7 as determined by BLAST, although they do not cluster into a gene family; “Unblast” = species-specific genes that are not observed in other species with e-values less than 1e-7 as determined by BLAST; and “Others” = orthologs that do not fit into the other categories. **b** Shared and unique gene families in *C. vestalis*, *D. collaris, N. vitripennis* and *A. mellifera* are shown in the Venn diagram. **c** Comparison of the distributions for identity values of orthologous genes in *C. vestalis*, *D. collaris* and *A. mellifera*. **d** Microsynteny in *C. vestalis* and *D. collaris* determined by tracking the gene positions. In addition to *C. vestalis* and *D. collaris*, species names and ordinal affiliations for the arthropods in the data set are: *Anopheles gambiae* (Diptera), *Apis mellifera* (Hymenoptera), *Bombyx mori* (Lepidoptera), *Copidosoma floridanum* (Hymenoptera), *Cimex lectularius* (Hemiptera), *Camponotus floridanus* (Hymenoptera), *Ceratosolen solmsi* (Hymenoptera), *Danaus plexippus* (Lepidoptera), *D. melanogaster* (Diptera), *Diadegma semiclausum* (Hymenoptera), *Lasioglossum albipes* (Hymenoptera), *Microplitis demolitor* (Hymenoptera)*, Plutella xylostella* (Lepidoptera), *Nasonia vitripennis* (Hymenoptera), *Pediculus humanus* (Phthiraptera), *Tribolium castaneum* (Coleoptera), and *Tetranychs urticae* (Trombidiformes)

We compared orthologous gene pairs identified in *C. vestalis* and *D. collaris* to 8 other hymenopteran species, 8 other insect species in diverse orders, and 1 mite species (*Tetranychus urticae*) in the order Trombidiformes (Fig. 3a). Over 85% of the genes in each of the wasp species we sequenced were orthologous to genes in one or more other species. The number of single-copy genes in *C. vestalis* and *D. collaris* was 659 (4.9%) and 665 (3.8%), respectively. These two wasps contained more than 6000 many-to-many universal genes, which accounted for 37–47% of the total gene sets (Fig. 3a). The wasp gene sets showed a significantly higher proportion of many-to-many orthologues than single-copy genes, suggesting that duplication occurs more frequently in the universal orthologues than in insect-specific genes.

We constructed a phylogenetic tree with 262 universal single-copy orthologues using maximum likelihood methods. Consistent with prior analyses [26], our results supported that these two parasitoid wasps in the superfamily Ichneumonoidea diverged from the Aculeata (bees and ants) approximately 140 million years ago, and the braconid *C. vestalis* and the ichneumonid *D. collaris* diverged approximately 124 million years ago (Fig. 3a). In total, we identified 83ll gene families in *C. vestalis* and 9063 in *D. collaris*. The number of unique gene/gene families in *C. vestalis* and *D. collaris* was 474 and 1060, respectively (Fig. 3b). *C. vestalis* shared 258 gene families with *D. collaris* (Fig. 3b). The occurrence of the same gene families in different parasitoid species could be a consequence of a common adaptive pathway to parasitic lifestyle. *C. vestalis*, *D. collaris* and *A. mellifera* encoded a similar proportion (about 60%) of single-copy orthologous that shared amino acid identities (Fig. 3c). Based on ratio of syntenying genes, very high degrees of microsynteny were observed between *C. vestalis* and *D. collaris* orthologs (Fig. 3d, Additional file 1: Table S10 and S11), much more than that between *C. vestalis* and *A. mellifera, D. collaris* and *A. mellifera* (Additional file 1: Table S10 and S11), which indicated numerous chromosomal rearrangements have occurred in hymenopteran species since diverged from their last shared ancestor. The synteny shared between *C. vestalis* and *D. collaris* reflect more conserved genes maintained across these two species.

### Gene family expansions and gene losses

**Fig. 4:**
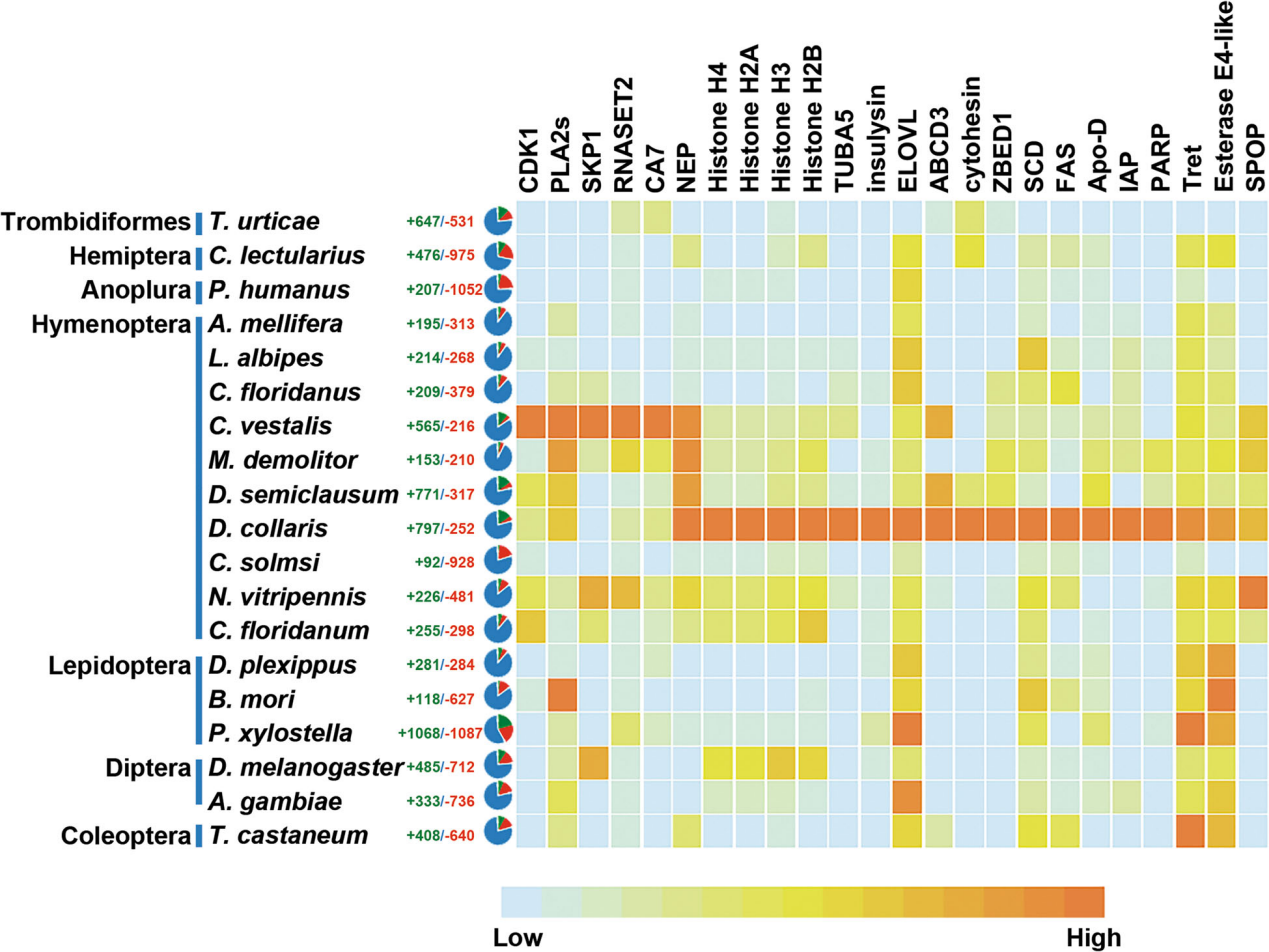
Gene families with significant expansions in *C. vestalis* and/or *D. collaris* when compared to select other arthropod species. Gene families in *C. vestalis* with significant expansions (*p* < 0.001, chi-square test) were: CDK1 (cyclin-dependent kinase 1), PLA2s (phospholipase A2-like), SKP1 (S-phase kinase-associated protein 1), RNASET2 (ribonuclease T2), and CA7 (carbonic anhydrase VII). Gene families with significant expansions in *D. collaris* were: four subfamilies of histone (H2A, H2B, H3, H4), FAS (fatty acid synthase), SCD (stearoyl-CoA desaturase (delta-9 desaturase)), ELOVL (elongation of very long chain fatty acids protein), TUBA5 (Tubulin alpha-5), ABCD3 (ATP-binding cassette sub-family D member 3), ZBED1 (zinc finger BED domain-containing protein 1), Apo-D (apolipoprotein D), IAP (apoptosis 1 inhibitor), PARP (poly [ADP-ribose] polymerase), Tret (trehalose transporter), and SPOP (speckle-type POZ protein). The number of NEP family was much higher in the four Ichneumonidae species when compared to other species in the Figure. The pie charts mean numbers of gene loss and gain in each genome: green means gene gain and red means gene loss. Slices with different colours represent numbers of orthologues in each expanded gene family. Light blue means the lowest number and orange means the highest number

**Fig. 5:**
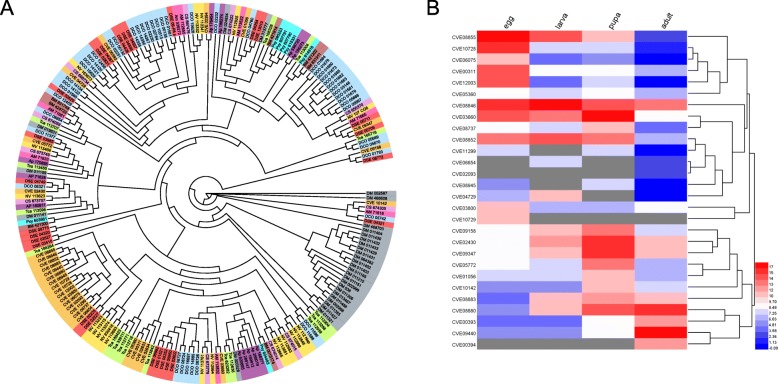
The phylogenetic relationships of neprilysins in insects and their expression levels in different stage of *C. vestalis*. **a** The phylogenetic relationships of neprilysins in insects. 216 neprilysins of 11 species (*A. mellifera*, *A. pisum*, *B. mori*, *C. solmsi*, *C. vestalis*, *D. collaris*, *D. melanogaster*, *D. semiclausum*, *N. vitripennis*, *P. xylostella*, *T. castaneum*) were used to construct the phylogenetic tree by maximum likelihood method. **b** Heatmap showing expression levels of all the NEP genes in different stage of *C. vestalis*. A total of 28 genes and two pseudugenes were found in *C. vestalis* genomes. Among the 28 NEP family genes in *C. vestalis*, more than half of them were highly expressed in parasitic periods (egg and larvae stages)

We used CAFÉ [27] to examine gene family expansions and contractions in this study*.* When compared to other arthropods, 30 gene families in *C. vestalis* and 65 in *D. collaris* exhibited significant expansions, while 23 gene families in *C. vestalis* and 3 in *D. collaris* were contracted (*P* < 0.05) (Additional file 3: Table S12). During further analysis of selected expanding gene families (*P* = 0) for analysis (Fig. 4), we found neprilysins (NEPs) was expanded in these two species and also other two hymenopteran species, *Diadegma semiclausum* and *M. demolitor* (Figs. 4 and 5a). We investigated the expression pattern of NEP genes from *C. vestalis* at different developmental stages via RNA-seq-based differential expression analysis (Fig. 5b). Among the 28 NEP genes in *C. vestalis*, more than half were highly expressed in eggs and larvae. Several gene families, such as *CDK1*, *SKP1*, *PLA2, RNASET2* and *CA7*, associated with developmental regulation showed expansions in *C. vestalis*. In *D. collaris*, we observed the expansion of histone genes and other genes encoding enzymes with functions in trehalose transport (*TRET*) and fatty acid metabolism, such as fatty acid synthase (*FAS*), stearoyl-CoA desaturase (*SCD*), and elongation of very long chain fatty acids protein (*ELOVL*) (Fig. 4).

We also observed that certain contracted gene families in *C. vestalis* were expanded in *D. collaris,* such as carboxylesterase, *SCD*, histone and ribonucleoside-diphosphate reductase beta chain. The expansion and contraction of the same gene family maybe, to some extent, reflect the different lifestyle of these two wasps. Wasps are carnivorous animals that evolved from a branch of herbivorous insects. It is reasonable that these two wasps lacked a number of enzymes required to synthesize nine essential amino acids (glutamate, histidine, isoleucine, leucine, lysine, methionine, phenylalanine, tryptophan, and valine), and two non-essential amino acids (arginine and tyrosine) (Additional file 1: Figure S3).

### Gene families associated with immunity

The *C. vestalis* and *D. collaris* genomes contained all components of the major insect immune pathways (Additional file 1: Table S13). However, comparisons to the other Hymenoptera (*Nasonia vitripennis, Apis mellifera*), Diptera (*Drosophila melanogaster, Anopheles gambiae*), and the host *P. xylostella*, we noticed the variation in the composition of certain immune gene families*.* For example, *C. vestalis, D. collaris,* and *A. mellifera* encoded smaller numbers of pattern recognition genes (peptidoglycan-recognition proteins (PGRPs), gram-negative bacteria binding proteins (GNBPs), galectins, fibrinogen-related proteins (FREPs) and C-type lectins than *N. vitripennis*, *D. melanogaster*, *A. gambiae*, and *P. xylostella*. The overall lower number of antimicrobial peptide genes (AMPs) were also found in *C. vestalis, D. collaris,* and *A. mellifera* larvae. However, these trends are not fully uniform given the greater number of *defensin* genes in *C. vestalis* (11) and *D. collaris* (7) relative to *N. vitiripennis* (5). In addition, 27 putative inhibitors of apoptosis (IAP) genes were identified in *D. collaris*, while other species contained only 3 to 7 (Additional file 1: Table S13).

Transcriptome analyses in *C. vestalis* showed the changes in the expression profiles of many immune-related genes during development (Additional file 4: Table S14); in particular, the expression levels of immune genes such as *defensin*, *serpin* and *C-type lectins* were significantly abundant in larvae and teratocytes of *C. vestalis.* We also determined that 8 of 27 *iap* genes in *D. collaris* were expressed in venom glands.

### Gene families associated with xenobiotic detoxification

*C. vestalis* and *D. collaris* together with other hymenopterans (*C. solmsi, N. vitripinnis* and *A. mellifera*) encoded less glutathione-S-transferases (GSTs) when compared with other arthropods (Additional file 1: Table S13). *C. vestalis* and *A. mellifera* also encoded less cytochrome P450s (CYPs) (Additional file 1: Table S15). In contrast, *D. collaris* encoded a very close number of P450s and carboxylesterases as *N. vitripinnis*, which were together broadly comparable to several other arthropods (Additional file 1: Table S13). Transcriptome analyses revealed that most of these detoxification enzyme genes were expressed in different life stages of *C. vestalis* (Additional file 4: Table S14). While no transcriptome data could be generated for different life stages of *D. collaris*, we speculate the detoxification enzyme genes identified in this species are likely expressed in similar stage-specific patterns to other hymenopterans.

## Discussion

More than 100 insect genomes have been sequenced during the last two decades [28], which provided valuable information to expand our understanding for the biodiversity of insect habits, behaviors and long-term evolutionary relationship. Yet, there are still many species with important roles in agriculture, which certainly are worth made thorough research. In this study, we report two phylogenetically related wasp genomes, *C. vestalis* and *D. collaris*, which are responsible for regulating the populations of a worldwide pest, *P. xylostella*. In spite of their strong genetic divergence, a small number of common features indicate that the genome of the wasps is, in certain ways, shaped by endoparasitism. This knowledge can be useful in revealing genomic convergence of parasitic wasps associated with the same host and the convergence to endoparasitic lifestyle.

The whole genome sequencing generated the draft genome of *C. vestalis* (178 Mb) and *D. collaris* (399 Mb). A total set that contained 11,278 and 15,328 protein-coding genes for *C. vestalis* and *D. collaris*, respectively, was predicted using evidence and de novo prediction methodology. Over 85% of the genes in *C. vestalis* and *D. collaris* were orthologous to genes in one or more other species. The number of unique gene/gene families in *C. vestalis* and *D. collaris* was 474 and 1060, respectively, and *C. vestalis* shared 258 gene families with *D. collaris.* Based on ratio of syntenying genes, very high degrees of microsynteny were observed between *C. vestalis* and *D. collaris* orthologs. When compared to other arthropods, 30 gene families in *C. vestalis* and 65 in *D. collaris* exhibited significant expansions, while 23 gene families in *C. vestalis* and 3 in *D. collaris* were contracted.

The gene gains and losses of *C. vestalis* and *D. collaris*, in some cases, reflected their shared life history as endoparasites and in other cases are unique to particular species. Certain gene families with predicted functions in development, nutrient acquisition from hosts, and metabolism have expanded, while genes required for biosynthesis of some amino acids and steroids have been lost as a potential consequence of these resources being available from their shared host, *P. xylostella*. Both species encode the highest number of NEPs thus far reported in arthropod genomes. NEPs are metalloproteases in the M13 peptidase family, which in vertebrates degrade several peptide hormones [29, 30] and amyloid beta [31, 32]. Recent studies also implicate NEPs in inhibiting coagulation of vertebrate blood through inactivation of fibrinogen and suppressing melanisation, a response regulated by the phenoloxidase (PO) cascade [33, 34]. Interesting, NEP is a major component of the parasitoid *Venturia canescens* virus like particles inducing protection of parasitoid eggs against encapsulation [35, 36]. Given its diversity function in immunity, we speculate that NEP expansion in *C. vestalis* and *D. collaris* could probably reflect a conserved role in the Ichneumonidae but not exclude the possibility that the expansion of this gene family is involved in evasion of host immune defenses. The expanding gene families in *C. vestalis*, *CDK1* and *SKP1* may involve in cell cycle progression, signal transduction and transcription, while *PLA2, RNASET2* and *CA7* are enzymes that catalyze a number of different biochemical reactions [37, 38]. However, the biological significance of expanding gene families associated with developmental regulation in *C. vestalis* is unclear, because they could contribute to either wasp physiology or the altered physiology of hosts. In *D. collaris,* the expansion of histone genes potentially reflects the rapid development of this species [39] and the need to quickly produce sufficient amount of protein to coat the genome when replicated during the S phase of every cell cycle, and it is also consistent with the increase in rRNA copy number we found. Meanwhile, the expansion of encoding enzymes with functions in trehalose transport (TRET) and fatty acid metabolism properly suggests their roles in exploring and using of host nutrients by *D. collaris* [40].

The *C. vestalis* and *D. collaris* genomes contained all components of the major insect immune pathways, but the gene numbers are varied in the composition of certain immune gene families. The overall lower number of immune related genes probably reflects the reduced risks of pathogen exposure in the environments where larval-stage endoparasitoids (hosts) and *A. mellifera* (colonies) reside [41] relative to the microbial-rich environments where ectoparasitic *N. vitripennis* larvae (carrion), *D. melanogaster* larvae (decaying fruit)*,* and *P. xylostella* larvae (cruciferous plants) develop. The significantly abundant expression profiles of many immune-related genes, such as *defensin*, *serpin* and *C-type lectins* in larvae and teratocytes of *C. vestalis* revealed by transcriptome analyses indicated the need to protect immunosuppressed hosts from secondary bacterial infections [42]. Same abundant expression profiles of given *iap* genes in *D. collaris* venom glands indicated these IAPs could be involved in the functions of venom, maybe inhibit the apoptosis of venom gland cell to continuously produce venom proteins in a longer time, or they could be delivered into host cells to interfere pupal development of hosts [43, 44].

Xenobiotic detoxification involves the conversion of lipid-soluble substances to water-soluble, extractable metabolites [45]. The process of xenobiotic detoxification is primarily affected by CYPs, carboxylesterases and GSTs in insect, which are known to vary between species as a function of taxon and life history [46]. Parasitoids of herbivores have also been suggested to exhibit relatively poor capabilities in metabolizing xenobiotics [47]. The overall greater number of detoxification genes in *D. collaris* relative to *C. vestalis* also suggests a higher capacity to detoxify xenobiotics. This could reflect a lower capacity by *P. xylostella* pupae to detoxify xenobiotics than larvae, which has been selected for greater investment in xenobiotics metabolism in *D. collaris*. It is also possible the larger number of P450s and carboxylesterases encoded by *D. collaris* have functions outside of xenobiotic detoxification given the roles of these enzymes in other physiological processes. Differences in the total inventory of gene families likely reflect a combination of ancestry, since each wasp species resides in different families or subfamilies of the Ichneumonoidea, and life history associated with each species exhibiting differences in the life stage of *P. xylostella* they preferentially parasitize.

## Conclusion

We presented the annotated genomes of the two endoparasitoid wasps *C. vestalis* and *D. collaris* that parasitized the same host *P. xylostella* using Illumina and Pacbio sequencing platforms. These data will be a fundamental resource for developing new methods of biological control for the diamondback moth and provide more insights into the evolutionary interactions between parasitic wasps and their host, i.e., the sequencing of the *C. vestalis* genomes provide the references needed for identifying *C. vestalis*-produced miRNAs and assessing their roles in parasitism of *P. xylostella* [48]. These genomes could also be used in comparative genomics analysis with the recently released genomes of other hymenopterans including closely related parasitic wasp species to study the origin and diversification of the parasitic lifestyle.

## Methods

### Insect rearing

Laboratory cultures of *C. vestalis* and *D. collaris* were maintained by parasitizing a laboratory culture of *P. xylostella*. Each wasp species was originally collected from the cabbage field (30.3009 N 120.0870 E) in Hangzhou, China. The culture of *P. xylostella* was also established from field-collected material, and was subsequently maintained by feeding larvae on cabbage grown at 25 °C, 65% relative humidity and a 14 h light:10 h dark photoperiod. Adult wasps were fed a 20% (w/v) honey solution. Each wasp species had been reared continuously for 5 years, spanning more than 100 generations, as inbred lines before the onset of this study.

### Genome sequencing

A whole genome shotgun strategy was used to sequence the genomes of *C. vestalis* and *D. collaris* using the Illumina HiSeq 2000 platform (Illumina, San Diego, CA, USA). Long-Read Single Molecule Real-Time (SMRT) sequencing was also used for sequencing *C. vestalis* and *D. collaris* genomes performed on PacBio Sequel platform (Pacific Biosciences, Menlo Park, CA, USA) with P6 polymerase binding and C4 chemistry kits. DNA from a single wasp was insufficient for all sequencing runs. We thus extracted DNA from 2000 *C. vestalis* pupae*,* and 1000 adults from *D. collaris* using Qiagen DNA extraction kit. However, the use of inbred laboratory cultures ensured low levels of intraspecific sequence variation. To avoid sequence context bias, we constructed libraries with different insert sizes for HiSeq sequencing, including 2 paired-end sequencing libraries (170 bp, 500 bp) and 4 mate-pair libraries (2 kbp, 5 kbp, 10 kbp, and 20 kbp) for *C. vestalis*, and 3 paired-end sequencing libraries (170 bp, 500 bp, 800 bp) and 4 mate-pair libraries (2 kbp, 5 kbp, 10 kbp, and 20 kbp) for *D. collaris*. While for PacBio sequencing, we constructed library of 20 kbp using the standard protocol. In total, we obtained 36.10 Gb of raw data for *C. vestalis*, and 65.91 Gb of raw data for *D. collaris*.

### Transcriptome sequencing

Samples were collected from four developmental stages (eggs, 2nd instar larvae, pupae, and adult females), teratocytes and venom glands of *C. vestalis* and venom glands of *D. collaris* for transcriptome sequencing. To avoid sample contamination by host material, wasp eggs and larvae were dissected from hosts and washed extensively in Petri dishes using TNH-FH medium (HyClone, Logan, UT, USA) before total RNA extraction. Teratocytes and venom glands were collected as previously described [21, 42]. Total RNA was isolated from the whole body using TRIzol reagent (Ambion, Foster City, CA, USA). RNA sequencing libraries were constructed using the Illumina mRNA-Seq Prep Kit (Illumina, San Diego, CA, USA). Oligo (dT) magnetic beads were used to purify mRNA molecules with poly (A). The mRNA was fragmented and was used as template, and random hexamers were used as anchor primers in the first-strand cDNA synthesis. The second-strand DNA was synthesized with DNA polymerase I to create double-stranded cDNA fragments. Double stranded cDNA was end repaired using T4 DNA polymerases and A-tailed using Klenow fragment lacking exonuclease activity. Illumina sequencing adapters were then added to the ends of double-stranded cDNAs and size selected by gel electrophoresis. Purified DNAs were then amplified by PCR followed by generation of 200 bp paired-end libraries that were sequenced using Illumina HiSeq 2000 platform.

### Small RNA libraries sequencing

We isolated 20 2nd instar larvae and of *C. vestalis*, teratocytes from 200 parasitized *P. xylostella* larvae and 20 3rd instar larvae of *D. collaris* from the parasitized *P. xylostella* pupae followed by washing each sample in TNM-FH medium three times. Total RNA was isolated using TRIzol reagent (Ambion, Foster City, CA, USA) followed by enrichment of 18-30 nt small RNAs using the PAGE method [49]. Small RNA sequencing libraries were constructed using TruSeq Small RNA Library Preparation Kits (Illumina, San Diego, CA, USA). Library sequencing was performed by Illumina HiSeq 2000. MicroRNA genes were inferred by miRdeep2 software against the Rfam database of release 11.0 [50].

### Estimation of genome size

K-mer refers to an artificial sequence division of K nucleotides iteratively from sequencing reads. A raw sequence read with L bp contains (L-K + 1) k-mers if the length of each k-mer is K bp. The frequency of each k-mer can be calculated from the genome sequence reads. K-mer frequencies along the sequence depth gradient follow a Poisson distribution in a given dataset. The genome size, G, is defined as G = K_num/K_depth, where the K_num is the total number of k-mers and K_depth is the frequency occurring more frequently than others. Short insert size libraries (170 bp and 500 bp for *C. vestalis* and 500 bp for *D. collaris*) were used to estimate the genome sizes with k-mer set as 17.

### Genome assemblies

To ensure the reliability of genome assembly, several types of reads were filtered. The filtering criteria was as following: (1) Reads from short insert-size libraries having an ‘N’ over 2% of its length and the reads from large insert-size libraries having an ‘N’ over 5% of its length of *C. vestalis*. (2) Reads from both insert-size libraries having an ‘N’ over 2% of its length of *D. collaris*. (3) Reads from short insert-size libraries having more than 30% bases with quality ≤7 and reads from large insert-size libraries having more than 40% bases with quality ≤7 of *C. vestalis*. Reads from short insert-size libraries having more than 40% bases with quality ≤7 and reads from large insert-size libraries having more than 60% bases with quality ≤7 of *D. collaris*. (4) Reads with more than 10 bp from the adapter sequence (allowing no more than two mismatches). (5) Small insert size paired-end reads that overlapped ≥10 bp between the two ends. (6) Two paired-end reads that were identical (and thus considered to be the products of PCR duplication). (7) Reads having k-mer frequency < 4 after correction (to minimize the influence of sequencing errors) of *D. collaris*.

The long read SMRT sequencing data was corrected using CANU (http://canu.readthedocs.org/) with default parameters [51]. After that, the corrected reads were used to extend the scaffolds assembled by SOAPdenovo V2.04, and the result was polished using Quiver (http://www.pacbiodevnet.com/Quiver/).

After these filtering steps 25.55 Gb (127.78×) from *C. vestalis* and 49.19 Gb (120.86×) from *D. collaris* were assembled using to SOAPdenovo V2.04 [24]. Reads from short insert size libraries (ranging from 170 to 800 bp) were split into different optimized sizes of k-mers to construct *de Bruijn* graphs and then merged into contigs by k-1 non-mismatching overlap for two k-mers. We tested different k-mer for each species to define the optimal K-mer for assembly. Based on the N50 and N90, the best assembly was achieved when K was set as 29-mer for *C. vestalis* and *D. collaris*. The paired-end information was subsequently used to link contigs into scaffolds from short insert sizes to long insert sizes. Gap close software KGF V1.19 and GapCloser V1.10 were used to fill gaps of *C. vestalis* and *D. collaris*. To assess assembly quality, high quality reads satisfying filtering criteria were aligned using SOAP2 with less than 3 mismatches. This yielded 95.9% (89.2× data) and 91.8% (63.7× data) coverage for the *C. vestalis* and *D. collaris* assemblies, respectively. Reads from the transcriptome data sets were assembled by SOAPdenovo2 with a k-mer size of 25. The quality of the assembly was checked using Benchmarking Universal Single-Copy Orthologs BUSCO v3.0.2 [25], using insectdbV9 as lineage dataset and reference.

### Repeat annotations

Tandem Repeats Finder (TRF) was used to search tandem repeats in each assembled genome [52]. Transposable elements (TEs) were predicted in the assemblies by homology searching against RepBase, using RepeatProteinMask and RepeatMasker [53] with default parameters.

### Gene prediction and functional annotation

We predicted gene sets in the assembled genomes using evidence (homology-based and transcriptome-based) and de novo prediction methodology. For homology-based prediction, *A. mellifera*, *D. melanogaster*, *N. vitripennis*, and *Tribolium castaneum* proteins were mapped onto the *C. vestalis* and *D. collaris* genomes using tblastn software [54]. Then, gene models were identified by mapping the homologous protein sequences against the tblastn hits detected in these two genomes using Genewise [55]. RNA-Seq data were mapped to the genome using TopHat [56], and transcriptome-based gene structures were obtained by cufflinks [56] (http://cufflinks.cbcb.umd.edu/). For de novo prediction, Augustus [57] and Genscan [58] were used to predict coding genes using appropriate parameters. GLEAN (http://sourceforge.net/projects/glean-gene/) was used to merge the gene sets, removing all genes with sequences less than 50 amino acids as well as those that only had de novo support. Finally, homology-based, de novo derived, and transcript gene sets were merged to form a comprehensive and non-redundant reference gene set. Gene functions were assigned based on the best match derived from each annotation by Blastp against the SwissProt and TrEMBL [59] databases. We annotated motifs and domains using InterPro [60] by searching against publicly available databases, including Pfam, PRINTS, PROSITE, ProDom, and SMART. Gene Ontology [61] information was retrieved from InterPro. We also mapped the reference genes to KEGG [62] pathway maps by searching KEGG databases and found the best hit for each gene.

### Identification of orthologues and synteny

Similarities and differences among *C. vestalis* and *D. collaris* genes were assessed by best reciprocal hit of protein sequences using BLASTP with e-values < 0.01 between any two pairs of species defined as orthologous counterparts. The similarity of genes was indicated as a density plot of aligned ratio and identity derived directly from Blastp. The amino acid identity of single-copy orthologous between *C. vestalis* and *D. collaris* was about 60% (Fig. 3a). Syntenic blocks between *C. vestalis* and *D. collaris* were identified based on the orthologous gene order as described above. Specifically, syntenic blocks were defined as at least 3 orthologous counterparts that are both clustered (not interrupted by more than 5 genes) and located in continuous loci in a single scaffold for each species in a given pair of species.

### Phylogenetic tree and divergence time

Coding sequences and protein data of *C. vestalis*, *D. collaris*, 8 other hymenopteran species including the publicly available genomes from four species of parasitic Hymenoptera (*N. vitripennis* (family Pteromalidae), *Ceratosolen solmsi* (Agaonidae), *M. demolitor* (family Braconidae), and *Camponotus floridanus* (family Encyrtidae) and one genome for *Diadegma semiclausum* (Hellen) (family Ichneumonidae), which is also a larval-pupal parasitoid of the diamondback moth *P. xylostella*, generated by Illumina technology in our laboratory, 8 other insect species in diverse orders (*Anopheles gambiae* (Diptera), *Bombyx mori* (Lepidoptera), *Cimex lectularius* (Hemiptera)*, Danaus plexippus* (Lepidoptera), *D. melanogaster* (Diptera), *P. xylostella* (Lepidoptera), *Pediculus humanus* (Phthiraptera), and *Tribolium castaneum* (Coleoptera), and 1 mite species (*Tetranychus urticae*) in the order Trombidiformes were downloaded from Ensembl. For genes with alternative splice variants, the longest transcripts were selected. We used Treefam [63] to define a gene family as a group of genes that descended from a single gene in the last common ancestor [64]. Phylogenetic trees were constructed from universal single-copy orthologues using maximum likelihood methods. The BRMC approach was used to estimate the species divergence time using the programme MULTIDIVTIME [65], which was implemented using the Thornian Time Traveller (T3) package (ftp://abacus.gene.ucl.ac.uk/pub/T3/).

### Gene family analysis

Coding sequences and protein data of the above 18 insects and one mite (*T. urticae*) were used as database. For genes with alternative splice variants, the longest transcripts were selected. We used Treefam [63] to define a gene family as a group of genes that descended from a single gene in the last common ancestor [64]. We used CAFÉ [27] to identify gene family expansions and contractions in this study.

### Statistical analyses

No statistical methods were used to predetermine sample sizes. Experiments were not randomized. The investigators were not blinded to allocation during experiments or outcome assessment.

## Supplementary information

**Additional file 1: Figure S1.** Seventeen-k-mer estimation of *C. vestalis* and *D. collaris* genome size. (A) The genome size of *C. vestalis* was estimated to be 203 Mb based on reads from 170 bp and 500 bp insertsize libraries. (B) The genome size of *D. collaris* was estimated to be 408 Mb based on reads from 170 bp and 500 bp insert size libraries. **Figure S2.** GC content and sequencing depth of *C. vestalis* (A) and *D. collaris* (B). 10 Kb non-overlapping sliding windows for *C. vestalis*, and 20 Kb non-overlapping sliding windows for *D. collaris* were used to calculate the GC content and average depth among the windows. **Figure S3.** Comparison of amino acids biosynthesis between *C. vestalis* and *P. xylostella*. Three colors were used to show the difference between the two species on the pathway map of biosynthesis of amino acids (ko01230). Permission to use this pathway map image was kindly granted by the KEGG curators [61]. **Table S1.** Summary statistics of whole-genome sequencing data of *C. vestalis* and *D. collaris.* **Table S2.** Summary statistics of filtered data of *C. vestalis* and *D. collaris.* **Table S3.** Statistics of the genome assembly of *C. vestalis* and *D. collaris.* **Table S4.** Transcriptome sequence map to the genome assemblies of *C. vestalis* and *D. collaris.* **Table S5.** General statistics of predicted protein-coding genes for *C. vestalis.* **Table S6**. General statistics of predicted protein-coding genes for *D. collaris.* **Table S7.** Statistics of function annotation for *C. vestalis* and *D. collaris.* **Table S8.** TE statistics for *C. vestalis*, *D. collaris* and other insect genomes. **Table S10.** Microsynteny of *C. vestalis* and *D. collaris* genomes. **Table S11.** Statistics of syntonic regions of *C. vestalis* and *D. collaris* genomes compare to other insects. **Table S13.** Comparisons of the immune-related genes among several arthropod species. **Table S15.** Comparisons of the detoxification genes among several arthropod species.

**Additional file 2: Table S9.** miRNAs in *C. vestalis* and *D. collaris.*

**Additional file 3: Table S12**. Expanded and reduced gene families in *C. vestalis* and *D. collaris* relative to other arthropods.

**Additional file 4: Table S14.** Expression level of immune and detoxification genes in *C. vestalis* and *D. collaris.*1. Kanehisa M, Goto S: KEGG: kyoto encyclopedia of genes and genomes. Nucleic Acids Res*.* 2000; 28(1): 27–30.

## Data Availability

The filtered data used for de novo assembly were deposited into NCBI under SRA: SRR6356304, SRR6356303, SRR6356306, SRR6356305, SRR6356302, SRR6356301 (Illumina HiSeq 2000) and SRR6513317 (PacBio Sequel), associated with BioProject PRJNA307296 and BioSample SAMN04378091 for *C. vestalis*; SRR6346143, SRR6346142, SRR6346141, SRR6346140, SRR6346146, SRR6346145, SRR6346144 (Illumina HiSeq 2000) and SRR6513331 (PacBio Sequel), associated with BioProject PRJNA307299 and BioSample SAMN04378100 for *D. collaris*. The genome assembly data have also been deposited at DDBJ/EMBL/GenBank under accession numbers LQNH00000000 (*C. vestalis*) and LQNJ00000000 (*D. collaris*). The version described in this paper is the first version. The annotated data are available at the Waspbase website: http://www.insect-genome.com/waspbase/download/download_content.php?downloads_type=Genome. The transcriptomic data are also available at the Waspbase website with a different directory: http://www.insect-genome.com/waspbase/download/download_content.php?downloads_type=Transcript.

## Abbreviations

BRMC: Bayesian relaxed molecular clock
BUSCO: Benchmarking Universal Single-Copy Orthologs
CvBV: *Cotesia vestalis* bracovirus
NEPs: Neprilysins
PDVs: Polydnaviruses
PO: Phenoloxidase
SMRT: Single Molecule Real-Time sequencing
TEs: Transposable elements
TRF: Tandem Repeats Finder
