# Supplementary material for: The genomes of two parasitic wasps that parasitize the diamondback moth
Source: BMC Genomics. 2019 Nov 21;20:893. doi: 10.1186/s12864-019-6266-0 (PMC6873472; doi:10.1186/s12864-019-6266-0)
Supplement: Supplementary file 1 — Additional file 1: Figure S1. Seventeen-k-mer estimation of C. vestalis and D. collaris genome size. (A) The genome size of C. vestalis was estimated to be 203 Mb based on reads from 170 bp and 500 bp insertsize libraries. (B) The genome size of D. collaris was estimated to be 408 Mb based on reads from 170 bp and 500 bp insert size libraries. Figure S2. GC content and sequencing depth of C. vestalis (A) and D. collaris (B). 10 Kb non-overlapping sliding windows for C. vestalis, and 20 Kb non-overlapping sliding windows for D. collaris were used to calculate the GC content and average depth among the windows. Figure S3. Comparison of amino acids biosynthesis between C. vestalis and P. xylostella. Three colors were used to show the difference between the two species on the pathway map of biosynthesis of amino acids (ko01230). Permission to use this pathway map image was kindly granted by the KEGG curators [61]. Table S1. Summary statistics of whole-genome sequencing data of C. vestalis and D. collaris. Table S2. Summary statistics of filtered data of C. vestalis and D. collaris. Table S3. Statistics of the genome assembly of C. vestalis and D. collaris. Table S4. Transcriptome sequence map to the genome assemblies of C. vestalis and D. collaris. Table S5. General statistics of predicted protein-coding genes for C. vestalis. Table S6. General statistics of predicted protein-coding genes for D. collaris. Table S7. Statistics of function annotation for C. vestalis and D. collaris. Table S8. TE statistics for C. vestalis, D. collaris and other insect genomes. Table S10. Microsynteny of C. vestalis and D. collaris genomes. Table S11. Statistics of syntonic regions of C. vestalis and D. collaris genomes compare to other insects. Table S13. Comparisons of the immune-related genes among several arthropod species. Table S15. Comparisons of the detoxification genes among several arthropod species. [file 12864_2019_6266_MOESM1_ESM.docx]

Supplemental Figures and Tables


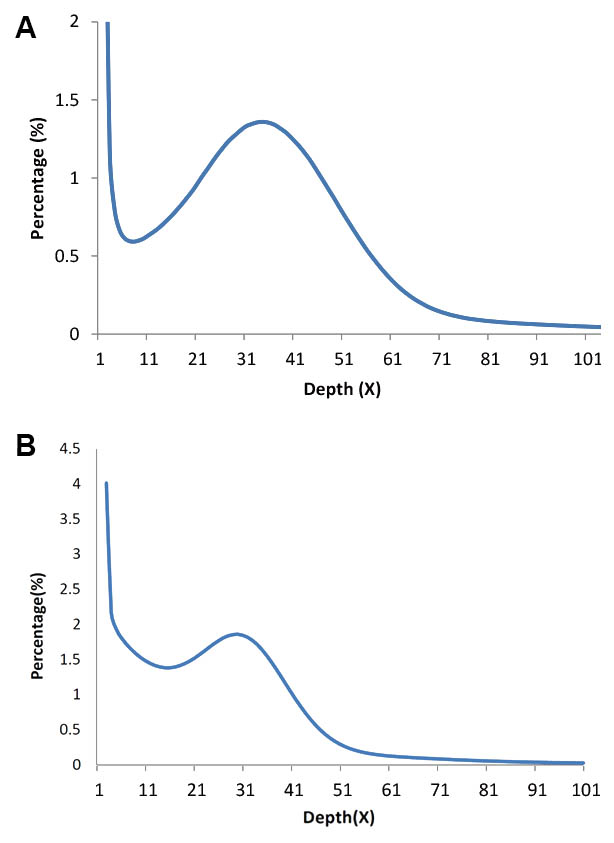


Figure S1. Seventeen-k-mer estimation of *C. vestalis* and *D. collaris* genome size. (A) The genome size of *C. vestalis* was estimated to be 203 Mb based on reads from 170 bp and 500 bp insert size libraries. (B) The genome size of *D. collaris* was estimated to be 408 Mb based on reads from 170 bp and 500 bp insert size libraries.


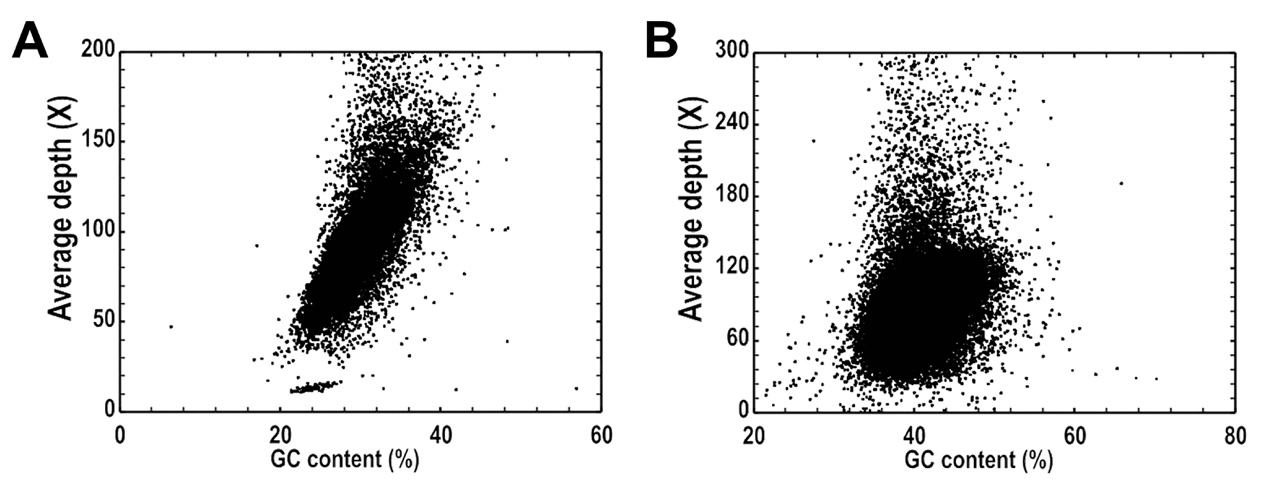
Figure S2. GC content and sequencing depth of *C. vestalis* (A) and *D. collaris* (B). 10 Kb non-overlapping sliding windows for *C. vestalis*, and 20 Kb non-overlapping sliding windows for *D. collaris* were used to calculate the GC content and average depth among the windows.


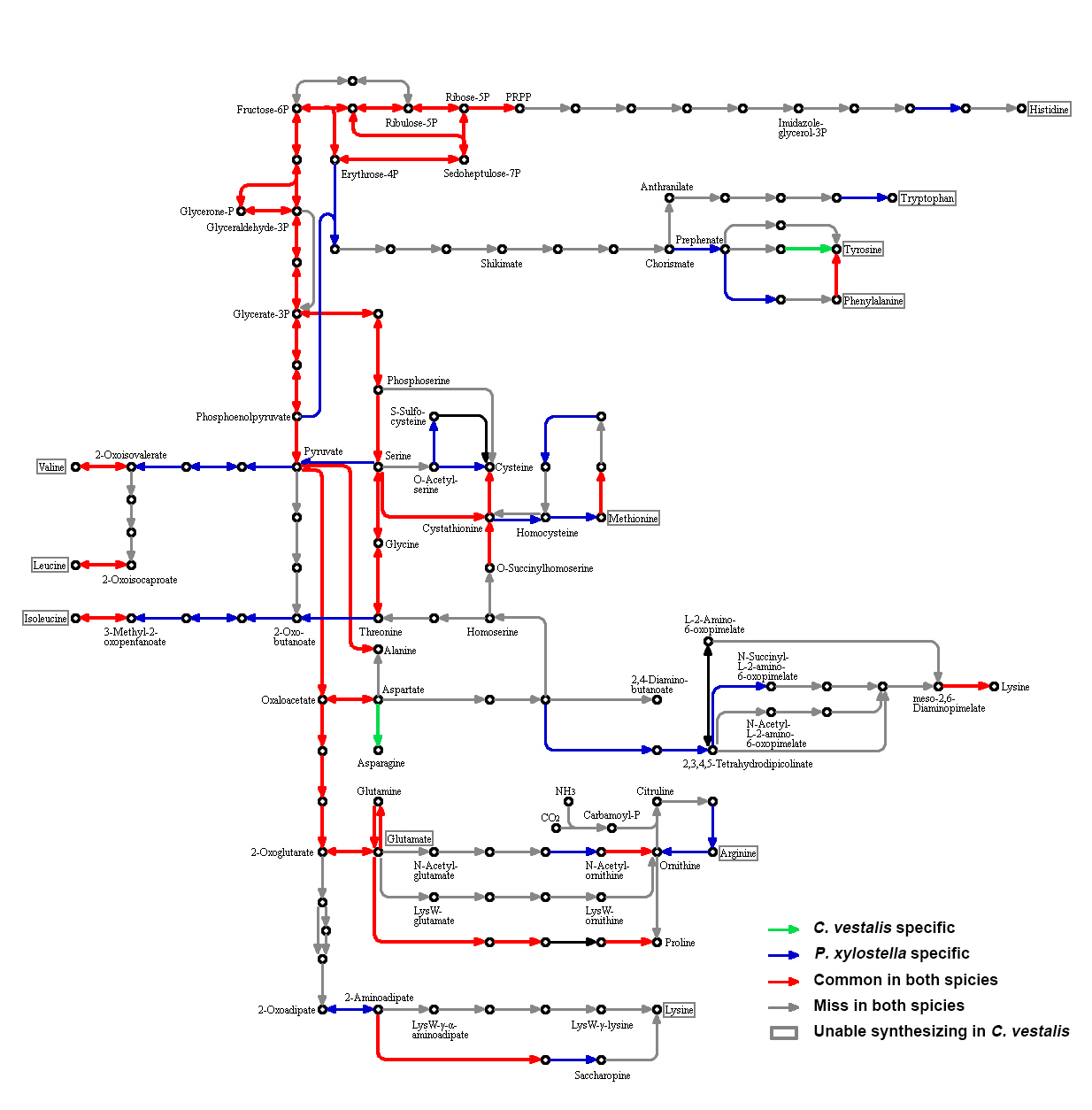


Figure S3. Comparison of amino acids biosynthesis between *C. vestalis* and *P. xylostella*. Three colors were used to show the difference between the two species on the KEGG pathway map of biosynthesis of amino acids (ko01230). Permission to use this pathway map image was kindly granted by the KEGG curators [61].

**Table S1. Summary statistics of whole-genome sequencing data of *C. vestalis* and *D. collaris***

| **Species** |  | Library Insert Size | Reads Length(bp) | Total Data(Gb) | Sequence Depth (X) |
| --- | --- | --- | --- | --- | --- |
| *C. vestalis* | Solexa | 170bp | 100 | 5.20 | 26.02 |
|  |  | 500bp | 100 | 3.57 | 17.87 |
|  |  | 2kb | 49 | 7.46 | 37.28 |
|  |  | 5kb | 49 | 7.26 | 36.28 |
|  |  | 10kb | 49 | 3.97 | 19.83 |
|  |  | 20kb | 49 | 4.60 | 22.99 |
|  | Solexa Total |  |  | 32.05 | 160.27 |
|  | Pacbio | 20kb |  | 4.05 | 20.23 |
|  | Total |  |  | 36.10 | 180.50 |
| *D. collaris* | Solexa | 200bp | 100 | 11.63 | 28.57 |
|  |  | 500bp | 100 | 9.38 | 23.05 |
|  |  | 800bp | 100 | 8.59 | 21.11 |
|  |  | 2kb | 49 | 15.64 | 38.43 |
|  |  | 5kb | 49 | 7.56 | 18.57 |
|  |  | 10kb | 40 | 4.96 | 12.19 |
|  |  | 20kb | 49 | 3.29 | 8.08 |
|  | Solexa Total |  |  | 61.05 | 150.0 |
|  | Pacbio | 20kb |  | 4.86 | 11.94 |
|  | Total |  |  | 65.91 | 161.94 |

**Table S2. Summary statistics of filtered data of *C. vestalis* and *D. collaris***

|  |  | Library Insert Size | Reads Length(bp) | Total Data(Gb) | Sequence Depth (X) |
| --- | --- | --- | --- | --- | --- |
| *C. vestalis* | Solexa | 170bp | 100 | 4.93 | 24.64 |
|  |  | 500bp | 100 | 3.31 | 16.56 |
|  |  | 2kb | 49 | 5.57 | 27.85 |
|  |  | 5kb | 49 | 3.45 | 17.23 |
|  |  | 10kb | 49 | 2.35 | 11.74 |
|  |  | 20kb | 49 | 2.33 | 11.66 |
|  | Solexa Total |  |  | 21.93 | 109.67 |
|  | Pacbio | 20kb |  | 3.62 | 18.11 |
|  | Total |  |  | 25.55 | 127.78 |
| *D. collaris* | Solexa | 200bp | 100 | 11.03 | 27.10 |
|  |  | 500bp | 100 | 8.23 | 20.22 |
|  |  | 800bp | 100 | 6.87 | 16.88 |
|  |  | 2kb | 49 | 11.89 | 29.21 |
|  |  | 5kb | 49 | 4.73 | 11.62 |
|  |  | 10kb | 40 | 2.70 | 6.63 |
|  |  | 20kb | 49 | 1.54 | 3.78 |
|  | Solexa Total |  |  | 46.99 | 115.45 |
|  | Pacbio | 20kb |  | 2.20 | 5.41 |
|  | Total |  |  | 49.19 | 120.86 |

**Table S3. Statistics of the genome assembly of *C. vestalis* and *D. collaris***

| **Species** |  | **Contig** | | **Scaffold** | |
| --- | --- | --- | --- | --- | --- |
|  |  | **Size (bp)** | **Number** | **Size (bp)** | **Number** |
| ***C. vestalis*** | **N90** | 5,991 | 4,490 | 59,784 | 249 |
|  | **N50** | 51,333 | 818 | 2,609,601 | 16 |
|  | **Longest** | 542,561 |  | 11,695,239 |  |
|  | **Total Size** | 171,707,290 |  | 178,546,584 |  |
|  | **Total Number (>100 bp)** |  | 39,668 |  | 32,746 |
|  | **Total Number (>2 kb)** |  | 6,820 |  | 1,437 |
| ***D. collaris*** | **N90** | 5,431 | 15,290 | 57,877 | 688 |
|  | **N50** | 25,941 | 4,123 | 1,030,362 | 107 |
|  | **Longest** | 205,319 |  | 5,788,798 | - |
|  | **Total Size** | 367,771,060 |  | 399,170,654 | - |
|  | **Total Number (>100 bp)** |  | 84,658 |  | 63,392 |
|  | **Total Number (>2 kb)** |  | 20,676 |  | 2,732 |

Table S4. Transcriptome sequence map to the genome assemblies of *C. vestalis* and *D. collaris*

| **Specie** | **Dataset** | **Number** | **Total Length**  **(bp)** | **Sequences**  **Covered by**  **Assembly** | **With >90% Sequence in**  **one Scaffold** | | **With >50% Sequence in**  **one Scaffold** | |
| --- | --- | --- | --- | --- | --- | --- | --- | --- |
|  |  |  |  |  | **Number** | **Percent** | **Number** | **Percent** |
| ***C. vestalis*** | All | 16,734 | 8,998,285 | 91.70% | 14,797 | 88.42% | 15,218 | 90.94% |
|  | >200bp | 14,446 | 8,603,601 | 93.51% | 13,050 | 90.34% | 13,390 | 92.69% |
|  | >500bp | 5,998 | 5,932,831 | 98.25% | 5,694 | 94.93% | 5,816 | 96.97% |
|  | >1000bp | 2,076 | 3,185,560 | 99.76% | 1,998 | 96.24% | 2,038 | 98.17% |
| ***D. collaris*** | All | 38,330 | 17,698,102 | 98.11% | 33,961 | 88.60% | 37,310 | 97.33% |
|  | >200bp | 38,330 | 17,698,102 | 98.11% | 33,961 | 88.60% | 37,310 | 97.33% |
|  | >500bp | 10,353 | 9,364,184 | 99.68% | 9,892 | 95.54% | 10,270 | 99.20% |
|  | >1000bp | 2,864 | 4,250,196 | 99.86% | 2,782 | 97.13% | 2,849 | 99.47% |

**Table S5. General statistics of predicted protein-coding genes for *C. vestalis***

| **Gene set** | | **Number** | **Average transcript length (bp)** | **Average CDS length (bp)** | **Average exon per gene** | **Average exon length (bp)** | **Average intron length (bp)** |
| --- | --- | --- | --- | --- | --- | --- | --- |
| *De novo* | AUGUSTUS | 11,266 | 4030.90 | 1505.55 | 4.75 | 317.04 | 673.64 |
|  | geneid | 12,493 | 4517.69 | 1504.74 | 5.00 | 301.18 | 753.96 |
| Homolog | *A. mellifera* | 10,902 | 4444.06 | 1325.03 | 4.06 | 326.68 | 1020.61 |
|  | *N. vitripennis* | 14,458 | 3168.21 | 1138.93 | 3.30 | 344.75 | 880.90 |
|  | *T. castaneum* | 12,563 | 3152.17 | 1126.41 | 3.30 | 341.17 | 880.15 |
|  | *D. melanogaster* | 9,180 | 3633.09 | 1155.09 | 3.54 | 326.05 | 974.55 |
| EST | | 20,408 | 3473.71 | 985.71 | 2.91 | 338.59 | 1301.76 |
| GLEAN | | 10,815 | 4816.18 | 1592.67 | 4.83 | 329.79 | 841.78 |
| RNA_seq | | 11,278 | 4863.78 | 1559.94 | 4.74 | 329.35 | 830.18 |

**Table S6. General statistics of predicted protein-coding genes for *D. collaris***

| **Gene set** | | **Number** | **Average transcript length (bp)** | **Average CDS length (bp)** | **Average exon per gene** | **Average exon length (bp)** | **Average intron length (bp)** |
| --- | --- | --- | --- | --- | --- | --- | --- |
| *De novo* | AUGUSTUS | 20,489 | 3689.26 | 1158.1 | 4.19 | 276.29 | 793.07 |
|  | geneid | 22,480 | 4038 | 1080.75 | 3.77 | 286.39 | 1066.21 |
| Homolog | *A. mellifera* | 13,407 | 6432.77 | 1245.36 | 4.52 | 275.44 | 1473.15 |
|  | *N. vitripennis* | 19,817 | 4375.72 | 1087.7 | 3.5 | 310.89 | 1315.94 |
|  | *T. castaneum* | 16,730 | 4229.92 | 1103.08 | 3.47 | 317.64 | 1264.53 |
|  | *D. melanogaster* | 10,642 | 5033.24 | 1070.04 | 3.99 | 268.08 | 1324.8 |
| EST | | 34,324 | 2075.36 | 492.73 | 1.86 | 265.57 | 1850.2 |
| GLEAN | | 15,414 | 5348.12 | 1294.6 | 4.39 | 294.68 | 1194.57 |
| RNA_seq | | 15,328 | 5449.75 | 1274.97 | 4.36 | 292.74 | 1174.03 |

|  |  | ***C. vestalis*** | | ***D. collaris*** | |
| --- | --- | --- | --- | --- | --- |
|  |  | **Number** | **Percent (%)** | **Number** | **Percent (%)** |
| Total | | 11,278 |  | 15,328 |  |
| Annotated | InterPro | 7,898 | 70.03 | 9,420 | 61.46 |
|  | GO | 6,444 | 57.14 | 7,634 | 49.80 |
|  | KEGG | 6,725 | 59.63 | 8,279 | 54.01 |
|  | Swissprot | 7,952 | 70.51 | 9,657 | 63.00 |
|  | TrEMBL | 9,584 | 84.98 | 11,697 | 76.31 |
| Unannotated | | 1,577 | 13.98 | 2,906 | 18.96 |

Table S7. Statistics of function annotation for *C. vestalis* and *D. collaris*

Table S8. TE statistics for *C. vestalis*, *D. collaris* and other insect genomes

| **Species** | **DNA (% of genome)** | **LINE (%)** | **SINE (%)** | **LTR (%)** | **Other (%)** | **Unknown (%)** | **Total (%)** |
| --- | --- | --- | --- | --- | --- | --- | --- |
| ***C. vestalis*** | 11,736,864  (6.57) | 4,732,971  (2.65) | 382,064  (0.21) | 14,234,765  (7.97) | 11,194  (0.01) | 19,805,303  (11.09) | 43,052,119  (24.11) |
| ***D. collaris*** | 27,127,319  (6.80) | 14,817,630  (3.71) | 1,238,331  (0.31) | 76,294,605  (19.11) | 35,401  (0.01) | 49,041,523  (12.29) | 148,111,143  (37.10) |
| ***D. semiclausum*** | 16,517,560  (6.32) | 5,553,267  (2.12) | 443,292  (0.17) | 38,273,272  (14.64) | 1,337  (0.00) | 8,894,627  (3.40) | 66,244,517  (25.33) |
| ***L. albipes*** | 46,604,764  (13.64) | 7,595,887  (2.22) | 54,414  (0.02) | 27,785,828  (8.13) | 1,645  (0.00) | 31,156,356  (9.12) | 91,180,752  (26.69) |
| ***P. xylostella*** | 38,095,301  (9.67) | 51,536,938  (13.08) | 15,313,451  (3.89) | 37,181,193  (9.44) | 57  (0.00) | 18,561,207  (4.71) | 130,580,857  (33.14) |
| ***N. vitripennis*** | 26,170,738  (8.87) | 13,698,632  (4.64) | 150,887  (0.05) | 27,606,078  (9.36) | 3,838  (0.00) | 15,364,911  (5.21) | 66,259,859  (22.45) |
| ***C. solmsi*** | 11,311,239  (4.07) | 2,824,099  (1.02) | 38,753  (0.01) | 7,293,952  (2.62) | 34,085  (0.01) | 3,602,732  (1.30) | 16,307,257  (5.87) |
| ***P. humanus*** | 7,698,964  (6.95) | 4,107,285  (3.71) | 296,167  (0.27) | 4,157,756  (3.75) | 201,506  (0.18) | 517,904  (0.47) | 8,504,594  (7.677) |
| ***D. melanogaster*** | 4,306,649  (2.55) | 11,592,141  (6.87) | 16,601  (0.01) | 20,136,524  (11.93) | 696,218  (0.41) | 5,204,638  (3.08) | 41,585,537  (24.65) |
| ***A. mellifera*** | 3,728,195  (1.63) | 746,542  (0.33) | 44,465  (0.02) | 1,820,345  (0.80) | 4,689  (0.00) | 1,896,072  (0.83) | 7,304,535  (3.20) |

Table S10. Microsynteny of *C. vestalis* and *D. collaris* genomes

| **Organism** | **Species** | **Orthology genes** | **Number of blocks** | **Block length** | **Rate of genome (%)** | **N50 of blocks** | **Genes in blocks** | **Rate of genes (%)** |
| --- | --- | --- | --- | --- | --- | --- | --- | --- |
| **Cve_Dco** | **Cve** | 7,303 | 1,360 | 57,373,860 | 30.28 | 87,618 | 5,103 | 39.38 |
|  | **Dco** | 7,303 | 1,360 | 133,369,065 | 33.41 | 244,898 | 6,557 | 38.90 |
| **Cve_Ame** | **Ame** | 5554 | 1473 | 81,438,984 | 35.63 | 163,304 | 5456 | 6.89 |
|  | **Cve** | 5554 | 752 | 91,956,255 | 51.5 | 2,838,746 | 7281 | 8.72 |
| **Ame_Dco** | **Ame** | 6202 | 1722 | 86,627,381 | 37.9 | 140,134 | 5810 | 7.34 |
|  | **Dco** | 6202 | 1400 | 103,808,709 | 39.7 | 209,646 | 6774 | 6.97 |

Cve: *C. vestalis*, Dco: *D. collaris*, Ame: *A. mellifera*.

Table S11. Statistics of syntonic regions of *C. vestalis* and *D. collaris* genomes compare to other insects

| **Subject_Query** | **Size** | **Synteny** | **%** | **Size** | **Synteny** | **%** |
| --- | --- | --- | --- | --- | --- | --- |
| **Cve_Dco** | 178,546,584 | 69,978,021 | 17.53 | 399,170,654 | 46,042,780 | 25.79 |
| **Cve_Ame** | 178,546,584 | 54,845,672 | 30.72 | 228,564,734 | 52,308,421 | 22.89 |
| **Cve_Dme** | 178,546,584 | 5,901,668 | 3.31 | 168,736,537 | 4,642,704 | 2.75 |
| **Dco_Ame** | 399,170,654 | 79,400,175 | 19.89 | 228,564,734 | 47,539,969 | 20.80 |
| **Dco_Dme** | 399,170,654 | 9,825,973 | 2.46 | 168,736,537 | 5,509,681 | 3.27 |

Cve: *C. vestalis*, Dco: *D. collaris*, Ame: *A. mellifera*, Dme: *D. melanogaster*.

**Table S13. Comparisons of the immune-related genes among several arthropod species**

| Gene family | Cve | Dse | Dco | Nvi | Ame | Dme | Aga | Pxy |
| --- | --- | --- | --- | --- | --- | --- | --- | --- |
| **Recognition** |  |  |  |  |  |  |  |  |
| PGRP | 4 | 3 | 4 | 11 | 4 | 13 | 7 | 9 |
| GNBP/βGRP | 2 | 1 | 4 | 2 | 2 | 3 | 7 | 18 |
| Galectins | 2 | 5 | 3 | 2 | 2 | 6 | 8 | 4 |
| FREPs (Fibrinogen-Related Proteins) | 2 | 3 | 4 | 1 | 2 | 14 | 61 | 2 |
| C-type lectins | 8 | 13 | 17 | 28 | 10 | 37 | 25 | 7 |
| Scavenger receptor | 26 | 35 | 30 | 19 | 13 | 23 | 21 | 15 |
| **Signaling** |  |  |  |  |  |  |  |  |
| **Toll Pathway** |  |  |  |  |  |  |  |  |
| Spz | 4 | 4 | 5 | 6 | 7 | 5 | 6 | 5 |
| Toll Receptor | 7 | 7 | 5 | 6 | 5 | 9 | 10 | 9 |
| MyD88 | 1 | 1 | 1 | 1 | 1 | 1 | 1 | 0 |
| Tube | 0 | 1 | 1 | 1 | 1 | 1 | 1 | 1 |
| Pelle | 2 | 1 | 1 | 1 | 1 | 1 | 1 | 3 |
| Cactus | 1 | 2 | 1 | 2 | 3 | 1 | 1 | 1 |
| TRAF6 | 1 | 1 | 2 | 1 | 1 | 1 | 1 | 2 |
| **IMD pathway** |  |  |  |  |  |  |  |  |
| IMD | 1 | 1 | 1 | 1 | 1 | 1 | 1 | 1 |
| FADD | 1 | 1 | 1 | 1 | 1 | 1 | 1 | 1 |
| Dredd (caspase 8) | 1 | 1 | 1 | 1 | 1 | 1 | 1 | 1 |
| TAK1 | 1 | 1 | 1 | 1 | 1 | 1 | 1 | 1 |
| Tab2 | 1 | 1 | 1 | 1 | 1 | 1 | 1 | 2 |
| IKK-β | 1 | 0 | 1 | 1 | 0 | 1 | 1 | 1 |
| IKK-γ | 1 | 1 | 1 | 1 | 1 | 1 | 1 | 2 |
| Relish | 1 | 1 | 1 | 1 | 2 | 1 | 1 | 3 |
| **JAK-STAT Pathway** |  |  |  |  |  |  |  |  |
| Domeless | 1 | 1 | 1 | 1 | 1 | 1 | 1 | 1 |
| Hopscotch | 2 | 1 | 1 | 1 | 1 | 1 | 1 | 1 |
| STAT | 1 | 1 | 1 | 1 | 1 | 1 | 2 | 2 |
| **Effectors** |  |  |  |  |  |  |  |  |
| PPO | 1 | 2 | 2 | 4 | 2 | 3 | 9 | 1 |
| TEP | 4 | 5 | 4 | 3 | 3 | 6 | 13 | 1 |
| LYS | 1 | 1 | 1 | 1 | 2 | 13 | 8 | 2 |
| AMP | 15 | 15 | 11 | 44 | 7 | 22 | 10 | 7 |
| **Others** |  |  |  |  |  |  |  |  |
| IAP(apoptosis) | 4 | 4 | 29 | 6 | 5 | 4 | 7 | 3 |
| Catalase(Peroxisome) | 2 | 2 | 1 | 1 | 1 | 2 | 1 | 13 |
| Peroxidase | 16 | 12 | 14 | 15 | 13 | 20 | 23 | 17 |
| SOD(Peroxisome) | 6 | 7 | 6 | 4 | 2 | 4 | 4 | 7 |
| Serpins | 16 | 12 | 15 | 12 | 7 | 28 | 17 | 26 |
| CLIP-domain Serine Protease | 3 | 3 | 4 | 17 | 28 | 65 | 49 | 13 |
| **Total** | **140** | **150** | **176** | **199** | **133** | **293** | **303** | **182** |

**Table S15. Comparisons of the detoxification genes among several arthropod species**

|  | Dco | Cve | Dse | Cso | Nvi | Api | Tca | Ame | Dme | Bmo | Pxy | Zne |
| --- | --- | --- | --- | --- | --- | --- | --- | --- | --- | --- | --- | --- |
| **P450** |  |  |  |  |  |  |  |  |  |  |  |  |
| cyp2 | 10 | 10 | 10 | 7 | 7 | 10 | 8 | 8 | 6 | 10 | 10 | 12 |
| cyp3 | 38 | 25 | 25 | 11 | 48 | 32 | 72 | 28 | 36 | 36 | 26 | 27 |
| cyp4 | 43 | 11 | 19 | 10 | 30 | 32 | 44 | 4 | 32 | 32 | 36 | 21 |
| Mitochondrial | 6 | 5 | 7 | 6 | 7 | 8 | 9 | 6 | 11 | 8 | 13 | 9 |
| Total | 97 | 51 | 61 | 34 | 92 | 82 | 133 | 46 | 85 | 86 | 85 | 69 |
| **GSTs** |  |  |  |  |  |  |  |  |  |  |  |  |
| Delta |  |  |  | 4 | 5 | 10 | 3 | 1 | 11 | 4 | 5 |  |
| Epsilon |  |  |  | 0 | 0 | 0 | 19 | 0 | 14 | 8 | 5 |  |
| Omega |  |  |  | 2 | 2 | 0 | 3 | 1 | 5 | 4 | 5 |  |
| Sigma |  |  |  | 4 | 8 | 6 | 7 | 4 | 1 | 2 | 2 |  |
| Theta |  |  |  | 0 | 3 | 2 | 1 | 1 | 4 | 1 | 1 |  |
| Zeta |  |  |  | 1 | 1 | 0 | 1 | 1 | 2 | 2 | 2 |  |
| total | 14 | 17 | 11 | 11 | 19 | 18 | 34 | 8 | 37 | 21 | 20 | 12 |
| **CCEs** |  |  |  |  |  |  |  |  |  |  |  |  |
| carboxylesterase | 22 | 8 | 7 | 17 | 41 | 30 | 49 | 24 | 35 | 69 | 13 |  |

Dco: *D. collaris*, Cve: *C. vestalis*, Dse: *D. semiclausum*, Cso: *C. solmsi*, Nvi: *N. vitripennis*, Api: *A. pisum*, Tca: *T. castaneum*, Ame: *A. mellifera*, Dme: *D. melanogaster*, Bmo: *B. mori*, Pxy: *P. xylostella*, Zne: *Z. nevadensis*.
